# Review of Antibiotic Resistance, Ecology, Dissemination, and Mitigation in U.S. Broiler Poultry Systems

**DOI:** 10.3389/fmicb.2019.02639

**Published:** 2019-11-15

**Authors:** Yichao Yang, Amanda J. Ashworth, Cammy Willett, Kimberly Cook, Abhinav Upadhyay, Phillip R. Owens, Steven C. Ricke, Jennifer M. DeBruyn, Philip A. Moore Jr.

**Affiliations:** ^1^Department of Crop, Soil, and Environmental Sciences, University of Arkansas, Fayetteville, AR, United States; ^2^Poultry Production and Product Safety Research Unit, United States Department of Agriculture, Agricultural Research Service (USDA-ARS), Fayetteville, AR, United States; ^3^Bacterial Epidemiology and Antimicrobial Resistance Research Unit, United States Department of Agriculture, Agricultural Research Service (USDA-ARS), Athens, GA, United States; ^4^Department of Poultry Science, University of Arkansas, Fayetteville, AR, United States; ^5^United States Department of Agriculture, Agricultural Research Service (USDA-ARS), Dale Bumpers Small Farms Research Center, Booneville, AR, United States; ^6^Department of Food Science and Center for Food Safety, University of Arkansas, Fayetteville, AR, United States; ^7^Department of Biosystems Engineering and Soil Science, University of Tennessee, Knoxville, Knoxville, TN, United States

**Keywords:** antibiotic resistant gene determinant, soil microbiome, broiler systems, One Health Approach, environmental dissemination

## Abstract

Since the onset of land application of poultry litter, transportation of microorganisms, antibiotics, and disinfectants to new locations has occurred. While some studies provide evidence that antimicrobial resistance (AMR), an evolutionary phenomenon, could be influenced by animal production systems, other research suggests AMR originates in the environment from non-anthropogenic sources. In addition, AMR impacts the effective prevention and treatment of poultry illnesses and is increasingly a threat to global public health. Therefore, there is a need to understand the dissemination of AMR genes to the environment, particularly those directly relevant to animal health using the One Health Approach. This review focuses on the potential movement of resistance genes to the soil via land application of poultry litter. Additionally, we highlight impacts of AMR on microbial ecology and explore hypotheses explaining gene movement pathways from U.S. broiler operations to the environment. Current approaches for decreasing antibiotic use in U.S. poultry operations are also described in this review.

## Introduction

### Antibiotic Use and History in U.S. Broiler Operations

Antimicrobial compounds and antibiotics in U.S. broiler (meat chicken) operations have widely been used to treat and prevent bacterial, protozoal, and fungal pathogens that sicken or kill birds, as well as promote growth (Chapman and Johnson, 2002; McEwen and Fedorka-Cray, 2002; Sneeringer et al., 2015). Considering, disease in broiler flocks can account for 20% loss of the gross value of production (Food and Agriculture Organization of the United Nations [FAO], 2013) antibiotics are important tools in poultry production. Continuous improvement in disease management and the establishment of government regulations has lead to staggering increases in poultry production efficiency [e.g., in 1965, 112 rearing days would produce a 1.13 kg chicken with a 4.7 feed conversion ratio (weight/feed intake); whereas current rearing periods are 42 days for a 2.7 kg chicken with a feed conversion ratio of 1.8] (National Chicken Council, 2015). Concurrent with production efficiency increases is consumption, as the average American now consumes 41 kg of broiler meat per year (National Chicken Council, 2018).

The first use of antibiotic drugs in poultry can be traced back to 1946 (Moore et al., 1946) and first resistance was reported in food animals by Starr and Reynolds (1951), with concerns about the development of resistance dating back to 1969 (Dibner and Richards, 2005). After the first cases of antibiotic resistant bacterial diseases in humans, recommendations were made for banning the use of antibiotics as growth promoters if drugs are also prescribed for use in human medicine (e.g., penicillins, tetracyclines, and sulfonamides; Swann et al., 1969). In a survey from 1995 to 2000, there was a substantial decline in the use of antibiotics in U.S. broiler operations (Food and Drug Administration [FDA], 2014). In another report released in 2011, it was estimated that 20–52% of broiler operations used antibiotics for production purposes not related to disease control. This report also found a long-term decline in antibiotic use in broiler production (Sneeringer et al., 2015). More recently, based on a report of antimicrobials sold or distributed for use in food-producing animals from the U.S. Food and Drug Administration (FDA), approximately 3,345,022 kg of antimicrobials were sold and used in the U.S. poultry industry in 2016; with 1,265,420 kg being “medically important” in human medical therapy (Food and Drug Administration [FDA], 2017). Among the most significant action that the FDA Center for Veterinary Medicine has taken, is to transition medically important antimicrobials that are used in the feed or drinking water of food-producing animals to veterinary oversight, and to eliminate the use of these products in animals for production purposes, such as for growth promotion (Guidance for Industry #213; Food and Drug Administration [FDA], 2013).

According to the World Health Organization (WHO), antimicrobial resistance (AMR) is defined by “an increase in the minimum inhibitory concentration of a compound for a previously sensitive strain” (World Health Organization [WHO], 2013). There are four general mechanisms that cause antibiotic resistance: target alteration, drug inactivation, decreased permeability, and increased efflux (Munita and Arias, 2016). It is still uncertain if resistance genes are a result of adaptation through chromosomal mutation (or gene shuffling), or through horizontal gene transfer (or the movement of genetic materials between different organisms), instead of vertical transmission of DNA from parent to offspring (Nesme and Simonet, 2015).

While specific links between antibiotic-use in animal agriculture and human health have been debated (Vaughn and Copeland, 2004), one contributing factor cited for the decline in antibiotic use is consumer demand for “antibiotic-free” chicken products. There is growing interest in sustainable food production and research is currently being conducted to identify antibiotic alternatives that could support healthy growth and provide defense against pathogenic microbes (Sneeringer et al., 2015; Gadde et al., 2017). Therefore, the broiler industry is now a new leader in management systems that seeks to eliminate the use of antibiotics for the entire broiler lifecycle. A comprehensive review of currently available compounds, their mechanism of action and advantage and disadvantages in applied broiler production is available from Gadde et al. (2017). A brief list of sample types, susceptibility to antibiotics, and mechanism of resistant can be found in Table 1.

**TABLE 1:** Sample sources, susceptibility to antibiotics, and mechanisms of potential AMR gene transfer to the environment.

**Sample sources**	**Susceptibility to antibiotics**	**Mechanisms of AMR gene transfer**	**References**
Poultry fecal waste	The study indicated that poultry samples showed a high prevalence of CTX-M cluster 9 and bla_TEM_.	Horizontal transfer of ARGs by Bacteriophages	Colomer-Lluch et al., 2011
Composted poultry manure	Poultry manure applications increased AMR genes in the rhizosphere, root endophyte, and phyllosphere, suggesting poultry manure may have an impact on lettuce resistomes.	No mechanism reported.	Zhang et al., 2019
Poultry litter	50% of these isolates were susceptible to ampicillin, 57% to erythromycin, 25% to tetracycline, 4% to chloramphenicol, 40% to kanamycin, 75% to streptomycin, 54% to tobramycin, and 4% to rifampicin.	Transformation and conjugation was reported as a mechanism for horizontal gene transfer between bacteria in poultry litter.	Sridevi Dhanarani et al., 2009
Poultry litter and soil	Out of the 13 antibiotics tested for *E. coli*, high (>70%) and similar (in the range of 10–15%) resistance against 7 antibiotics was observed in samples from both litter and agricultural soils where poultry litter applied.	No mechanism reported.	Bhushan et al., 2017
Poultry litter	The 86% of litter isolates (163 isolates in total) were resistant to more than one antibiotic.	No mechanism reported.	Furtula et al., 2013

Finally, the U.S. is the world’s largest poultry producer with over 9 billion broilers produced annually, with roughly 45% of broilers being produced in 4 mid-south states (Arkansas, North Carolina, Georgia, and Alabama). Poultry litter is a combination of bedding material, poultry excreta, spilled feed and feathers and is produced in significant quantities. By some estimates, nearly 13 million Mg (14 million tons) of broiler litter is produced on U.S. poultry farms annually (Moore et al., 1995; Gollehon et al., 2001). Consequently, large volumes of manure are produced in areas of concentrated poultry production, which serve as a valuable source of nutrients, but are also as possible sources of AMR bacterial populations in the environment (Thanner et al., 2016). Approximately 30–80% of the veterinary antibiotics administered to animals are excreted in manure and urine (Sarmah et al., 2006). Therefore, poultry litter-amended soil may serve as a non-point source for antibiotics that enter surface and ground waters via runoff and leaching. The goal of this review is to provide an update on the development and fate of antibiotic resistance genes (ARG) and bacteria in U.S. broiler poultry operations, and explore hypotheses explaining gene movement pathways to the environment. In the next section, resistance transmission and factors contributing to its development in poultry operations will be discussed as it relates to the soil microbiome.

#### Reservoirs and Transmission of AMR Bacteria and Genes From Farm-to-Field

**FIGURE 1:**
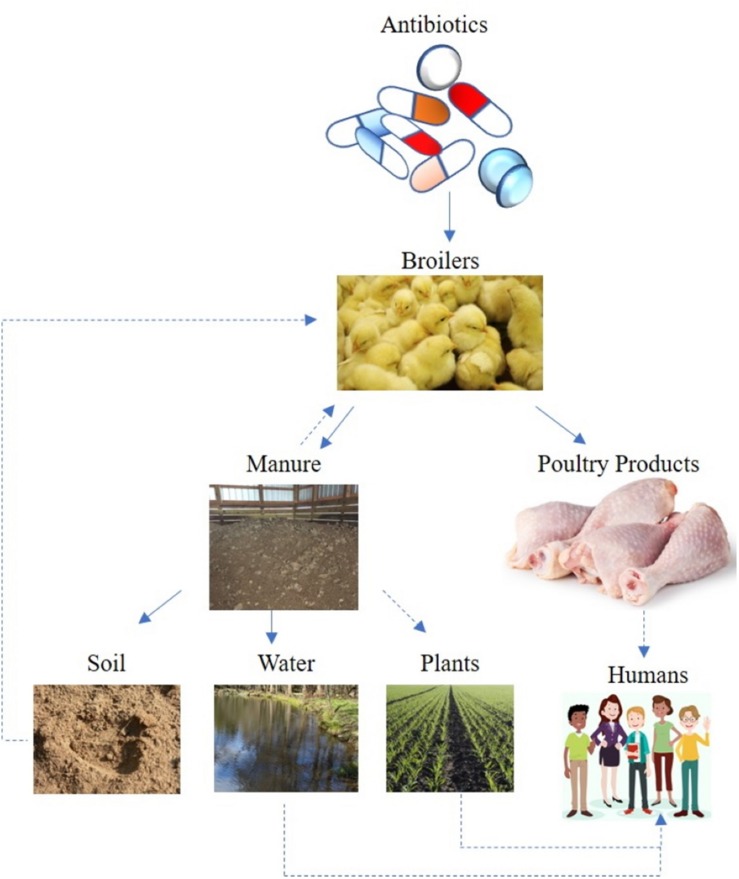
Potential AMR transmission route from broiler chicken antibiotic induction – to flock – to either poultry litter or meat products and to the soil-water environment. Solid lines suggest direct transmission, while dotted lines indicate indirect or possible transmission route.

Soils are an immense reservoir of microbiological diversity, considering a gram of soil may contain 10^6^–10^9^ bacterial cells of 10^3^–10^6^ different bacterial species (Girvan et al., 2003; Torsvik, 2011). Therefore, it is no surprise that the majority of antimicrobial compounds used in animal healthcare were originally isolated from the soil; namely bioactive compounds synthesized by bacteria (e.g., *Streptomyces* spp.) or fungi (Waksman and Woodruff, 1942). Consequently, the complex ecology of AMR can only be properly assessed by taking environmental reservoirs into account (Figure 1). In contrast to the strict clinical definition of resistance, which characterizes resistance phenotypes in isolated bacterial strains, the environmental resistome includes all ARG in the environmental, including ARG precursor genes and cryptic resistance genes (Nesme and Simonet, 2015). Recent research has identified ARG in diverse environmental samples ranging from pristine environments to agricultural soils (Demanèche et al., 2008; Allen et al., 2009; Cook et al., 2014). For these reasons, soil is a predominant reservoir for ARG determinants (ARGD, or determinants of resistance; Van Goethem et al., 2018). For example, AMR genes have been recovered from 30,000 years old permafrost samples, which suggests AMR is an ancient phenomenon, existing before antibiotic usages (D’Costa et al., 2011). Laboratory work also demonstrates that antibiotic resistant strains are very stable even in the absence of antibiotic selection pressure (Gibreel et al., 2005). Consequently, AMR development by pathogenic bacteria and/or commensal (or “friendly”) bacteria is a complex interaction and an evolutionary phenomenon.

Current research has focused on tracking the direction of gene transfer from environment to poultry and has important implications for future antibiotic resistance management and microbial ecology (Cook et al., 2014; Nesme and Simonet, 2015). Three research studies indicate it is probable that lateral resistance gene transfer is the primary pathway of gene acquisition from different environments, including that from soils to pathogenic bacteria genomes (Allen et al., 2009; Forsberg et al., 2012; Nesme et al., 2014). For example, Forsberg et al. (2012) evaluated resistant bacteria via functional metagenomic methods and determined that substantial amounts of resistance genes are shared between the soil and the gut microbiome and can transfer resistance to a previously susceptible *Escherichia coli* host. A shared resistome was also observed (Allen et al., 2009; Nesme et al., 2014) with metagenomics sequencing. These studies continue to emphasize the importance of environmental reservoirs of AMR in the emergence of novel clinical resistance (Nesme and Simonet, 2015).

While some research has not distinguished the direction of transfer (either through gene acquisition or through modulation), studies have shown that commensal (non-pathogenic) and pathogenic microorganisms share resistance genes with soil communities. Specifically, contact of antimicrobial compounds may stimulate bacterial stress response, which can result in increased mutation rates in co-dispersed bacteria, with co-selection amplifying this effect; thus allowing clustering of ARG (Yong-Guan et al., 2017). For example, DNA element class 1 integrons, which are assemblies of gene cassettes that allow bacteria to adapt and evolve through the expression of new genes, can capture and integrate foreign genes from the environment. This has played an important role in spreading antibiotic resistance from non-pathogenic bacteria to pathogenic bacteria in the environment (Yong-Guan et al., 2017). Next generation sequencing now indicates that a derivative of class 1 integrons can be found in every gram of feces and agricultural animals, with up to 10^23^ copies being released into the environment every day (Gillings, 2017). This is one example of the abundance and distribution of resistant genes, although more research is needed to identify anthropogenic AMR genes in the environment relative to baseline levels (Durso et al., 2016).

The pathogenic bacteria pathway from the animal through the environment is complicated and more longitudinal studies are needed to follow AMR genes through agricultural systems (Yang et al., 2019). These complex transmission routes of AMR bacteria and genes within animals and the environment make it difficult to identify the AMR reservoir and which reservoir poses an animal health. The current approach for assessing the reservoir of AMR bacteria and genes is to identify the indicator bacteria and analyze the level of AMR gene in farm animals (Thanner et al., 2016). With this research, descriptive metadata are needed that describes specific environments, which may reflect survivability and gene transfer. For example, the Terra Genome project includes soil information needed to evaluate terrestrial DNA which includes: (1) site description, (2) sampling description, (3) climate, (4) soil classification, and (5) soil analysis (Cole et al., 2010). These metadata should be important for pathogen viability, easy and inexpensive to obtain, and collected by established and standard methods. These metadata may provide a better understanding and potential mitigation strategies to minimize AMR dissemination.

### Sources of AMR Genes in the Environment

The role of the environment as a transmission route for bacterial pathogens has long been recognized, often associated with fecal contamination of water or organic fertilizer applications (Bengtsson-Palme et al., 2018). Depending on antibiotic properties, significant (e.g., up to 90%) amounts of veterinary antibiotics pass un-degraded through the animal gut to manure (Sarmah et al., 2006; Berendsen et al., 2015). Bacterial pathogens can be introduced to a flock via many routes, including feed, water, air, insects and other pests (Trampel et al., 2014; Mouttotou et al., 2017). Once introduced into a flock, pathogenic bacteria are excreted in the manure, and can survive in the litter (Chen and Jiang, 2014). Therefore, antibiotics, resistance genes, and microorganisms can be transferred from manure to soil (Cook et al., 2014; He et al., 2014). Following land application of poultry litter, antibiotics migrate from soil through runoff, leaching, and particle adsorbed runoff (Kay et al., 2004; Leal et al., 2013; Sun et al., 2013), potentially ending up in soil, surface water, and groundwater (He et al., 2014; Figure 1). Measuring antibiotics in a complex matrix, such as soil, is subject to technical limitations, and studies measuring veterinary pharmaceuticals in soil and water are reviewed elsewhere (Thiele-Bruhn, 2003; Dinh et al., 2011; Aga et al., 2016).

Several research efforts have been conducted for testing sources of AMR pathogenic bacteria and the transmission route from the chicken flock, to processing, and the larger environment (Berndtson et al., 1996; Berrang et al., 2001; Posch et al., 2006; Cook et al., 2014). Numerous routes have been suggested for the introduction of AMR pathogens into the chicken flock, such as horizontal gene transfer from an environmental source to the chicken flock (Krauland et al., 2009), or vertical transmission from breeder to progeny chicks (Pearson et al., 1996). Feed and water can also serve as potential reservoirs and transmit AMR pathogens from the environment to the chicken flock (Byrd et al., 1998; Perez-Boto et al., 2010). Although it is thought that cross-contamination of meat products can occur during the slaughter process (Berndtson et al., 1996; Berrang et al., 2001), there is limited information related to the transmission route from one part of contaminated meat to the whole retail product.

### Mechanisms of AMR Gene Transfer

Even though it has been suggested that there is a relationship between antibiotic usage in agricultural animals and AMR emergence, it does not mean that the usage of antibiotics is the only explanation for AMR prevalence. For example, AMR genes are carried by mobile genetic elements and can be transferred among distantly related bacteria from different phyla (Musovic et al., 2006). Plasmids and transposons can serve as the vehicle in horizontal gene transfer. Transposons, coding for antibiotic resistance, are able to cut AMR genes from one bacterial chromosome or a plasmid, and subsequently insert AMR genes into another chromosome or plasmid in other bacteria by the process of conjugation. Through this process, multiple AMR genes can be transferred among different bacteria; thus resulting in multi-drug resistance. Without antibiotic exposure, AMR genes may be able to persist long-term, such as VanA glycopeptide-resistant *Enterococci* (Johnsen et al., 2002). For example, van-resistant *Enterococci* can reportedly be stable after 1,000 generations in serial transfer broth cultures and gnotobiotic mice without antibiotic selection. The administration of antibiotics to farm animals, as a stressor to select AMR genes, is only one explanation and AMR can be driven by acquisition of mobile genes that existed in bacteria and evolved over time in the environment.

### Poultry Manure as a Reservoir for Resistant Genes

Approximately 6.9 kg per 1000 kg live weight per day is produced for a typical broiler operation, or 0.6–1.8 Mg per 1,000 broilers per flock (dry weight basis; American Society of Agricultural and Biological Engineers [ASABE] (2005); Moore, 2011), and there is concern that its land application may transport AMR microorganisms and genes to the environment, along with excreted drug residues from birds given antimicrobials, and residual disinfectants used in cleaning. For these reasons, AMR bacteria may be able to spread to the environment by application of litter to soils, which could possibly contribute to an increased frequency of horizontal gene transfer in the soil environment. Land application of poultry litter may also increase the diversity and dissemination of novel gene fragments among soil bacterial populations (Heuer et al., 2009). Research from Binh et al. (2009) indicated that the clinically relevant AMR gene, *aadA* (encoding resistance to streptomycin and spectinomycin), was introduced via poultry manure into soil. Cook et al. (2014) also evaluated AMR genes in land applied poultry litter and found that litter-borne AMR bacteria flourish following applications.

Typical antibiotic concentrations in manure range from 1 to 10 mg kg^–1^ (Kumar et al., 2005; Dolliver et al., 2007; Heuer et al., 2011), whereas soil and water concentrations range from trace to μg kg^–1^ or μg L^–1^, respectively (Thiele-Bruhn, 2003). In a comparison of indoor and free-range production systems, He et al. (2014) found that ARG in soil were positively correlated with antibiotic, metal, and nutrient concentrations. These data also suggest that both direct selection and co-selection mechanisms contribute to the suite of AMR genes detected. In the following section, authors discuss current approaches for decreasing the likelihood of AMR genes in U.S. broiler operations, as well as mitigation strategies for reducing AMR development.

#### Current Approaches for Decreasing AMR in Poultry Operations in the U.S.

The development and transmission of AMR determinants in microbial communities in poultry gastrointestinal tracts or on poultry products is a complex phenomenon fueled by a plethora of biotic and abiotic factors. Current approaches for decreasing AMR in poultry operations consist of coordinated multidisciplinary strategies aimed at developing new drugs and antibiotic alternatives and management approaches, and reducing total antibiotic usage (Food and Drug Administration [FDA], 2013). A brief description of each approach is provided below.

### Antibiotic Alternatives-Prebiotics, Probiotics, and Antimicrobial Compounds

Development of new antimicrobial drugs is a very labor intensive, time consuming and costly pursuit. More than 20 classes of antibiotics were identified from 1930 to 1962 (Coates et al., 2002). Since then, however, only a few classes of antibiotics have been approved (Butler and Buss, 2006). Other antimicrobial compounds such as antimicrobial peptides, peptidomimetics (Mojsoska and Jenssen, 2015), and virulence inhibitors (Mühlen and Dersch, 2015; Muhs et al., 2017) are being investigated for their efficacy against poultry pathogenic bacteria. Although found to be effective, their application in the industry would require significant industry-level testing and standardization.

Research is also currently being conducted to identify potent antibiotic alternatives that could provide both growth promotion for poultry and defense against microorganisms (Ricke, 2015; Upadhyaya et al., 2015a, b; Ricke, 2018). Prebiotics, probiotics, and antimicrobial compounds are the three major groups that are added to poultry water to reduce pathogenic bacteria colonization in the gut and subsequent contamination of poultry products. The efficacy of antibiotic alternatives on reducing *Campylobacter* colonization has been summarized in this review (Table 2). Prebiotics are defined as substrates that are selectively utilized by host microorganisms conferring a health benefit (Gibson et al., 2017). These beneficial effects could be through nutritional supplementation of beneficial microorganisms and/or through imparting resistance to pathogenic bacteria colonization. Fructans and galactans are examples of popular prebiotics with several research investigations highlighting their effect in enriching beneficial gut bacteria such as *Lactobacillus* and/or *Bifidobacterium* spp. (Bajury et al., 2017). With advances in microbiome research, our understanding of gut microbiota composition and substances that modify the microbiota has improved. This has expanded the prebiotic concept to include new compounds such as yeast-based products (Park et al., 2017) and dietary fibers (Ricke, 2015, 2018).

**TABLE 2:** The efficacy of antibiotic alternatives (phyto chemicals, probiotics, and probiotics and prebiotics) on reducing *Campylobacter* colonization and counts in broilers.

Phyto chemicals	Solis de los Santos et al. (2008; 2009; 2010) demonstrated that in feed supplementation of Caprylic acid, a medium chain fatty acid consistently reduced *Campylobacter* colonization in broiler chickens.
	Kollanoor Johny et al. (2010) previously reported the *in vitro* ability of thymol and carvacrol to inhibit both *Campylobacter jejuni* and *Salmonella* Enteritidis in chicken cecal contents.
	Results from Kollanoor Johny et al. (2012) suggest that Transcinnamaldehyde and Eugenol supplemented through feed could reduce *Salmonella* Enteritidis colonization in market-age chickens.
	Upadhyaya et al. (2013) revealed that antimicrobial wash with Eugenol or carvacrol rapidly inactivated *S*. Enteritidis on eggs to below detection limit at 32°C.
	Arsi et al. (2014) reported that in feed supplementation of plant extracts such as thymol or carvacrol reduced *Campylobacter* colonization in broiler chickens.
	Wagle et al. (2017a) demonstrated that supplementation of β-resorcylic acid in poultry feed for 14 days at 0.5 and 1% reduced *Campylobacter* populations in cecal contents by ∼ 2.5 and 1.7 Log CFU/g, respectively.
	Use of select doses of β-resorcylic acid showed significant reduction of *C. jejuni* on chicken skin and meat samples (Wagle et al., 2017b).
Probiotics	Aguiar et al. (2013) selected isolates for enhanced motility and the results from this study demonstrated that motility-enhanced isolates are more efficacious than unenhanced isolates in reducing *Campylobacter* colonization in broiler chickens.
	Arsi et al. (2015a) screened 116 isolates of probiotic strains and reported that six out of 116 strains reduced *Campylobacter* counts by at least 1–2 log.*in vivo.*
Probiotics and prebiotics	In a separate study, Arsi et al. (2015b) demonstrated that prebiotics did not consistently reduced *Campylobacter.* However, prebiotics (MOS) did significantly decrease the Campylobacter load when used in combination with *Probiotics* spp. (Arsi et al., 2015b).

Probiotics are live microorganisms, which when administered at adequate dosages, confer a health benefit on the host (World Health Organization [WHO], 2011). The major mechanisms by which probiotics act include competitive exclusion, improving barrier function, immunomodulation, and metabolic effects such as quorum sensing mitigation and virulence modulation in pathogenic bacteria (Oelschlaeger, 2010). In addition to their applications in human nutrition, probiotics are increasingly being supplemented in poultry feed for their health benefits. The commonly used genera include *Bifidobacterium, Bacillus, Lactobacillus*, and *Lactococcus.* Several probiotics have been reported to decrease the colonization of *Campylobacter* in the gastrointestinal track of broilers (Eeckhaut et al., 2016). This ability of probiotics to reduce poultry associated foodborne pathogenic bacteria could be due to their ability to promote beneficial gut bacteria that may exclude pathogens. For example, probiotic strains of human origin-*Lactobacillus rhamnosus* GG, *Propionibacterium freudenreichii* spp. *shermanii* JS, and *Lactococcus lactis* spp. *lactis* were found to attach to chicken intestinal mucus thereby reducing the binding and colonization of *Campylobacter* (Ganan et al., 2013). In addition to *Campylobacter*, several probiotic candidates have shown efficacy in reducing colonization of *Salmonella* spp. *in vitro* (Muyyarikkandy and Amalaradjou, 2017; Nair and Kollanoor Johny, 2017) and in poultry (Higgins et al., 2008).

Another management practice that could reduce the dissemination of AMR genes is the use of plant extracts. Plant-derived compounds represent a relatively safe, effective, and environmentally friendly source of antimicrobials. Plant extracts have been used in many cultures as food preservatives and dietary supplements for reducing spoilage and promoting growth. Due to their low cost, non-toxic nature, and antimicrobial efficacy, several phytochemicals are promoted as in-feed or in-water (nanoemulsion forms) supplements for reducing poultry pathogenic bacteria colonization. Extensive research in the last 2 decades has identified a plethora of compounds with antimicrobial efficacy (Gracia et al., 2016; Guyard-Nicodeme et al., 2016). Compounds such as caprylic acid (obtained from coconut oil), trans-cinnamaldehyde (from cinnamon bark), carvacrol (from oregano oil), and eugenol (from clove oil) have found to be effective in controlling *Salmonella* and *Campylobacter* in poultry (Kollanoor Johny et al., 2010, 2012; Arsi et al., 2014; Upadhyaya et al., 2015a, b). Medium chain fatty acids emulsion (caproic, caprylic, capric, and lauric acids) also reduce *Campylobacter* survival in drinking water and feed (Solis de los Santos et al., 2008, 2009; Solís de los Santos et al., 2010). Similar anti-*Campylobacter* efficacy has been reported with the addition of monocaprin emulsion (Thormar et al., 2006) thyme, and pine oil (Ozogul et al., 2015) in poultry feed. Research is still needed on how the use of these compounds may disrupt ARGD moment and fate in the environment.

### Management Approaches for Controlling AMR Development

Identifying feasible management practices is one of the objectives of the USDA Action Plan to control AMR development in animal agriculture (United States Department of Agriculture [USDA], 2014). Establishing good farm practices, maintaining proper hygiene, controlling vectors that transmit poultry pathogens, reducing stress in poultry during housing and transport, and identification of Critical Control Points during processing that contribute to AMR development are some of the key areas that hold promise and require detailed investigations. Scientifically, validated studies are required to test the effect of aforementioned factors on AMR development in poultry production and develop appropriate recommendations for the industry.

There is some evidence to suggest organic practices may also reduce the spread of AMR genes to the environment (Rothrock et al., 2016). For example, when comparing the numbers of infected hens from conventional and organic farms, hens from organically grown farms were less infected by *Campylobacter* than from conventional grown farms (Lestari et al., 2009; Kassem et al., 2017). *Campylobacter* isolated from organically grown hens exhibited significantly lower resistance to three antibiotics: ciprofloxacin, erythromycin, and tylosin (Kassem et al., 2017). However, the study from Noormohamed and Fakhr indicated that multidrug resistance existed in both organic and conventional farms (Noormohamed and Fakhr, 2014).Lestari et al. (2009) also provided differences of AMR patterns between conventional and organic chicken. Among 126 *Salmonella* isolates from conventionally and organically raised chicken carcasses obtained from retail stores in Louisiana, *Salmonella* isolates from organic chicken samples were susceptible to 11 of the antimicrobials, while isolates from conventional chickens were only susceptible to 4 antimicrobials (Lestari et al., 2009). However, it is still too early to conclude that organic chickens are less likely to harbor AMR than conventionally raised chickens.

## Concluding Remarks

Antibiotic resistance is a common ecological feature in soil, as is antibiotic production, therefore, AMR genes are ubiquitous and represent a reservoir of transferable genetic material. In addition, resistance is an advantageous trait for microorganisms surviving stressful environmental conditions. Only since the 1970s has it been realized that soils receiving poultry litter may be a major reservoir and transmission route for ARG. Thanks to advances in molecular biology, bioinformatics, and sequence data throughput, much more data are available on resistance genes, as well as the complex matrix that is soil and poultry litter. However, untangling the complexity of microbial ecology and environmental factors (e.g., particle size, pH, water availability, vegetation cover etc.) as it relates to transfer (transformation, conjugation or transduction) of genetic resistance is a multifaceted issue and widely considered a key challenge facing agriculture.

A major challenge facing microbiologists is to track dissemination of resistance genes in poultry production systems and identify reservoirs of resistance genes. Understanding factors that drive selection and dissemination of environmental antibiotic resistance, as well as mitigation strategies that will reduce the environmental dissemination of ARG. Future improvements in monitoring AMR movement in surface water from land-applied poultry litter will be critical to prevent the spread of resistance genes in the environment.

The pathogenic bacteria pathway from animals through the environment is complicated and more research is needed to follow AMR genes through these systems using the One Health Approach. Finally, owing to consumer demand for antibiotic-free meat products, much research has been done on promising antibiotic replacements (e.g., prebiotics, probiotics, and antimicrobial compounds). However, further research is needed on their efficacy and influence on AMR gene movement from farm-field.

## Author Contributions

YY, AA, KC, CW, and AU assisted in writing sections of the manuscript, as well as conceived of the outline and overall direction of this manuscript. PO, SR, JD, and PM provided final edits and direction on the current state of the literature, as well as guidance on soil and poultry microbiology.

## Disclaimer

Mention of tradenames or commercial products in this publication is solely for the purpose of providing specific information and does not imply recommendation or endorsement by the U.S. Department of Agriculture.

## Conflict of Interest

The authors declare that the research was conducted in the absence of any commercial or financial relationships that could be construed as a potential conflict of interest.

## Abbreviations

AMR: antimicrobial resistant
ARG: antibiotic resistant gene
ARGD: antibiotic resistant gene determinant
FDA: Food and Drug Administration
NARMS: National Antimicrobial Resistance Monitoring Systems
WHO: World Health Organization.
